# Single-Stage Endovascular Management of Concurrent Intracranial Aneurysms and Arterial Stenoses: Clinical Outcomes, Procedural Strategies, and Predictive Factors

**DOI:** 10.3390/brainsci15070744

**Published:** 2025-07-11

**Authors:** Marat Sarshayev, Shayakhmet Makhanbetkhan, Aiman Maidan, Roger Barranco Pons, Dimash Davletov, Abzal Zhumabekov, Mynzhylky Berdikhojayev

**Affiliations:** 1 National Hospital of the Medical Center of the Presidental Affairs Administration of the Republic of Kazakhstan, Almaty 050060, Kazakhstan; sarshayev_m@snh.kz (M.S.); makhanbetkhan_sh@snh.kz (S.M.); zhumabekov_ash@snh.kz (A.Z.); mynzhyl@gmail.com (M.B.); 2 National Centre for Neurosurgery, Astana 010000, Kazakhstan; 3 Al Qassimi Hospital, Dubai 25314, United Arab Emirates; rogerbarrancopons@gmail.com; 4 Atchabarov Scientific-Research Institute of Fundamental and Applied Medicine, Asfendiyarov Kazakh National Medical University, Almaty 050000, Kazakhstan; davletovdimash@gmail.com

**Keywords:** intracranial aneurysm, carotid artery stenosis, vertebral artery stenosis, endovascular treatment, single-stage intervention, subarachnoid hemorrhage, modified Rankin Scale, smoking, neurointervention, stroke prevention

## Abstract

Background: The coexistence of extracranial arterial stenoses and intracranial aneurysms presents a unique clinical dilemma. While staged interventions are traditionally preferred to reduce procedural risks, recent advances have enabled single-stage endovascular treatment. This study evaluates the clinical outcomes, procedural strategies, and predictive factors associated with such combined interventions. Methods: This retrospective study included 47 patients treated with single-stage endovascular procedures for concurrent extracranial stenosis and intracranial aneurysm between 2016 and 2024. Clinical, angiographic, and procedural data were collected. Outcomes were assessed using the mmodified Rankin Scale (mRS), and statistical analyses were performed to identify associations between clinical variables and functional outcomes. Results: Of the 47 patients, 85.1% achieved favorable outcomes (mRS 0–2) at ≥6-month follow-up. The most commonly treated arteries were the internal carotid artery (70.2%) and the middle cerebral artery (34%). Stent-assisted coiling or flow diversion was performed in 93.6% of aneurysm cases, while 91.5% underwent carotid or vertebral stenting. Lesion laterality (left-sided aneurysms, *p* = 0.019) and stenosis length (*p* = 0.0469) were significantly associated with outcomes. Smoking was linked to multiple stenoses (*p* = 0.0191). Two patients experienced major complications: one aneurysmal rebleed after stenting, and one intraoperative rupture. Conclusions: Single-stage endovascular treatment for patients with concurrent extracranial stenosis and intracranial aneurysm is technically feasible and clinically effective in selected cases. Lesion configuration, anatomical considerations, and individualized planning are critical in optimizing outcomes.

## 1. Introduction

The coexistence of extracranial arterial stenosis and intracranial aneurysm presents a complex therapeutic challenge. Historically, these lesions have been addressed separately, typically beginning with carotid endarterectomy or stenting, followed by delayed aneurysm treatment. However, this sequential approach can result in delays, increased procedural burden, and the risk of aneurysm rupture due to hemodynamic changes following revascularization [1,2,3,4,5].

In the era of modern endovascular techniques, single-stage interventions have emerged as a feasible and potentially safer option for selected patients. This strategy not only minimizes anesthesia exposure and hospital stay but also offers a streamlined solution for resource-limited settings, where repeated access procedures are economically burdensome [6,7,8,9].

Despite the increasing application of this approach, there is limited literature addressing patient selection, procedural sequencing, and outcome predictors in such cases. Concerns remain regarding the optimal order of intervention—whether to treat the stenosis first to improve access and reduce ischemic risk, or to secure the aneurysm first to prevent hemorrhagic complications exacerbated by reperfusion [10,11,12,13]. Additionally, few studies systematically evaluate the influence of anatomical configuration (ipsilateral vs. contralateral lesions), lesion length, or hemodynamic factors on functional recovery.

This study aims to assess the safety, feasibility, and clinical outcomes of single-stage endovascular treatment in patients with coexisting extracranial stenosis and intracranial aneurysms. We also propose a treatment algorithm based on real-world decision making and lesion-specific characteristics, contributing practical insights for individualized care planning.

## 2. Materials and Methods

This retrospective study was conducted at the National Hospital of the Medical Center of the Presidential Affairs Administration of the Republic of Kazakhstan in Almaty, Kazakhstan, and included patients treated between January 2016 and December 2024. Eligible patients had coexisting intracranial aneurysms and extracranial arterial stenoses (internal carotid artery [ICA] or vertebral artery [VA]) and underwent single-stage endovascular treatment.

### 2.1. Inclusion Criteria Were

• Age ≥ 18 years.
• Confirmed ≥70% extracranial ICA or VA stenosis based on digital subtraction angiography (DSA), CTA, or MRA, evaluated using NASCET criteria.
• At least one saccular or fusiform intracranial aneurysm identified via CTA or DSA.
• Either symptomatic ischemia (e.g., TIA, infarct on DWI) or aneurysm-related symptoms (e.g., headache, SAH).
• Available pre- and post-procedural imaging and clinical records.

Patients with previous intracranial or extracranial interventions, contraindications to dual antiplatelet therapy, or incomplete follow-up were excluded.

### 2.2. Imaging and Decision Protocol

Pre-treatment imaging included brain MRI with DWI to assess ischemic burden and CTA or DSA to evaluate aneurysm morphology, perfusion patterns, and vessel patency. The decision to treat both lesions in one session was made by a multidisciplinary neurovascular team. In patients with contralateral lesions or ruptured aneurysms, treatment sequencing was individualized. When perfusion through the stenotic segment was severely compromised, revascularization was prioritized. In other cases, aneurysm embolization preceded stenting to minimize rupture risk.

### 2.3. Procedure

The protocol has remained consistent since 2016; slight updates were implemented in 2020 after the introduction of newer stents. Endovascular procedures were performed under general or local anesthesia using transfemoral access. Aneurysms were treated via coiling, stent-assisted coiling, or flow diversion. Arterial stenoses were treated with balloon angioplasty and/or stenting. In all ICA cases, distal embolic protection devices were employed. Device selection was based on anatomical characteristics and operator preference.

### 2.4. Periprocedural Management

All patients without subarachnoid hemorrhage (SAH) received dual antiplatelet therapy (aspirin and clopidogrel) at least 5 days before the procedure. In SAH cases, DAPT initiation was delayed or minimized based on hemorrhage severity and urgency of intervention. Intravenous heparin was administered intraoperatively. Postprocedural antiplatelet regimens were adjusted per stent type and patient risk profile.

### 2.5. Data Collection and Outcomes

Demographic, clinical, radiological, and procedural data were retrospectively collected from electronic health records and imaging archives. Variables included age, sex, comorbidities (hypertension, diabetes, ischemic heart disease, smoking status), aneurysm characteristics (location, size, rupture status), and stenosis features (side, degree, length, symptomatic status).

Procedural details such as stent type, access route, and sequence of interventions were also documented. Modified Rankin Scale (mRS) scores were assessed at baseline and at follow-up by an independent neurologist. The PHASES score was used for aneurysm risk stratification. The primary outcome was a favorable functional outcome at last follow-up, defined as mRS 0–2. Secondary outcomes included periprocedural complications, 30-day mortality, and 30-day re-admission. Complications were categorized as hemorrhagic, ischemic, or technical.

### 2.6. Statistical Analysis

Descriptive statistics were used for baseline data. Associations between clinical variables and multiple stenoses or aneurysms were assessed using Fisher’s exact test. Logistic regression was employed to evaluate the association between smoking and multiple stenoses. The relationship between anatomical factors (e.g., lesion laterality, stenosis length) and functional outcome (mRS 1–2 vs. mRS 3–6) was also tested. A *p*-value < 0.05 was considered statistically significant. Analyses were performed using SPSS v26 (IBM Corp., Armonk, NY, USA). Regression analysis was used for exploratory purposes; findings should be interpreted cautiously due to small sample size.

### 2.7. Ethics Statement

This study was approved by the Institutional Ethics Committee (approval #7, dated 12 December 2024). Patient data were anonymized in compliance with the Declaration of Helsinki.

## 3. Results

A total of 47 patients (mean age: 67.6 ± 5.9 years; 21 males [44.7%], 26 females [55.3%]) were included. Most patients (95.7%) underwent elective procedures; only two patients (4.3%) presented with ruptured aneurysms requiring urgent intervention. Common presenting symptoms included headache (38.3%), ischemic stroke-related deficits (44.7%), and sensorineural symptoms such as hearing loss (4.3%). One patient presented with Millard–Gubler syndrome.

### 3.1. Patient Characteristics and Vascular Lesion Profile

In total, 17 patients (36.2%) were active smokers, 11 (23.4%) had diabetes mellitus, and 38 (80.9%) had stage 3 arterial hypertension. Ischemic heart disease was present in 34 patients (72.3%). Aneurysms were most frequently located in the middle cerebral artery (MCA), anterior cerebral artery (ACA), and intracranial ICA segments. The majority of stenoses involved the cervical ICA (57.4%) and vertebral artery (23.4%). Ipsilateral aneurysm–stenosis pairs were observed in 29 patients (61.7%). Patient characteristics can be seen in Table 1.

### 3.2. Risk Factors and Lesion Characteristics

Smoking was significantly associated with the presence of multiple stenoses (75.0% in smokers vs. 25.0% in non-smokers; *p* = 0.0191, OR = 0.131; 95% CI: 0.023–0.750). Logistic regression confirmed this association, with an odds ratio (OR) of 0.131 (95% CI: 0.023–0.750), indicating that non-smokers were significantly less likely to have multiple stenotic lesions. Other vascular risk factors, including hypertension, diabetes, ischemic heart disease, and obesity, showed no significant association with either multiple stenoses or multiple aneurysms (*p* > 0.05 for all; Table 2).

### 3.3. Treatment Strategy

The procedural strategy was tailored based on anatomical configuration and clinical presentation. Most cases involved flow diversion or stent-assisted coiling for aneurysm treatment, followed by angioplasty and carotid or vertebral artery stenting. In cases with high-grade ICA stenosis impeding microcatheter navigation, stenting was performed first. In morphologically unstable aneurysms (e.g., wide-neck or high-flow lesions), embolization was prioritized. Dual antiplatelet therapy was used in all elective cases pre- and post-procedurally. No elective patient experienced a deterioration in clinical status postoperatively.

Common devices included Protege (n = 17), ULTIMASTER (n = 8), and CASPER (n = 2). No ischemic periprocedural events were recorded.

### 3.4. Functional Outcomes and Predictive Factors

At the final follow-up (≥6 months), favorable outcomes (defined as mRS 0–2) were observed in 40 of 47 patients (85.1%). Modified Rankin Scale scores remained stable across the follow-up period for all patients, with no evidence of neurological deterioration. Among the cohort, seven patients (14.9%) had mRS scores of 3–5, indicating moderate to severe disability. No patient experienced mortality or re-admission within 30 days post-procedure.

Notably, left-sided aneurysms were significantly associated with favorable outcomes; all 14 patients with left-sided lesions achieved mRS scores of 1–2 (*p* = 0.019). However, no significant associations were found between outcome and aneurysm-bearing artery (*p* = 0.0761), side of extracranial stenosis (*p* = 0.7017), type of stenotic artery (*p* = 0.9497), or ipsilateral versus contralateral lesion configuration (*p* = 0.3108) (Table 3).

Among the anatomical predictors, stenosis length showed a statistically significant association with functional outcome (*p* = 0.0469), with longer stenotic segments correlating with worse mRS scores. In contrast, neither aneurysm size (*p* = 0.4291) nor stenosis severity as measured by percent narrowing (*p* = 0.2588) demonstrated predictive value for clinical outcome (Table 4).

These findings underscore the importance of lesion morphology—particularly stenosis length—over traditional size-based metrics in predicting recovery. The observed left-side laterality advantage warrants further investigation, potentially reflecting procedural or anatomical factors that influence outcome.

### 3.5. Complications

Two notable complications were recorded. One patient with a ruptured MCA aneurysm and an ipsilateral critical ICA stenosis developed a subarachnoid hemorrhage after stenting, requiring external ventricular drainage and a decompressive craniectomy; the mRS at 6 months was 5. Another patient experienced an intraoperative aneurysm rupture during coiling (non-ipsilateral), which was managed with intra-arterial nimodipine due to catheter-induced vasospasm; the patient had an mRS of 4 at 4 months.

### 3.6. Case Examples

Case 1: Simultaneous Treatment of Left MCA Aneurysm and Vertebral Artery Stenosis.

**Figure 1:**
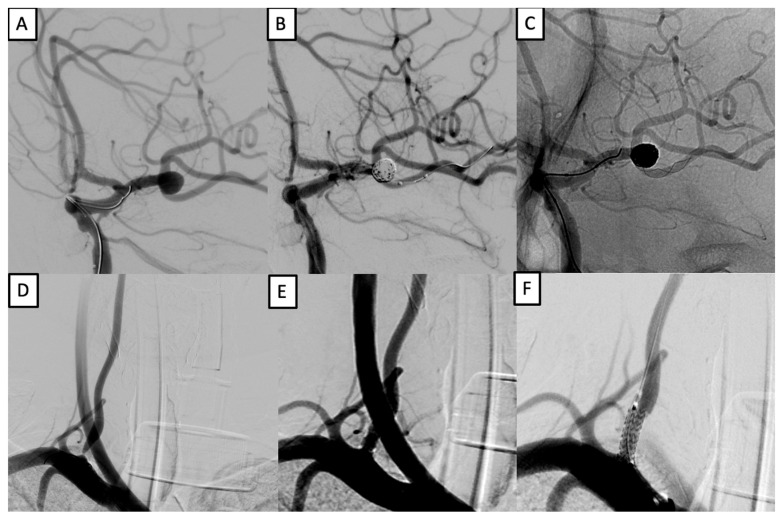
Simultaneous treatment of left MCA aneurysm and right vertebral artery stenosis. (**A**–**C**): balloon-assisted coiling with LEO stent (2.5 × 18 mm) of a left MCA bifurcation aneurysm. (**D**–**F**): stenting of right vertebral artery V1 segment using an Ultimaster 3.5 × 12 mm stent.

Patient #13 was a 38-year-old male presenting with recurrent occipital headaches, tinnitus, visual disturbances, and transient ischemic attacks. Imaging revealed a saccular aneurysm at the left middle cerebral artery (MCA) bifurcation and an 80% stenosis of the V1 segment of the right vertebral artery. Comorbidities included grade 3 arterial hypertension, impaired glucose tolerance, and grade 1 obesity. A single-stage endovascular procedure was performed. The aneurysm was occluded using balloon-assisted coiling with a LEO stent (2.5 × 18 mm) via the C-stenting technique. In the same session, vertebral artery stenosis was treated with an Ultimaster stent (3.5 × 12 mm). The postoperative course was uneventful, and the patient achieved an mRS of 1 at six months (Figure 1).

Case 2: Multivessel Stenosis and Dual Aneurysm Treatment Following SAH.

Patient #12 was a 57-year-old male with a history of smoking and hypertension who initially presented with a subarachnoid hemorrhage (SAH) and a ruptured left ICA aneurysm, treated with coiling. Follow-up imaging showed incomplete occlusion, and additional saccular aneurysms at the left MCA bifurcation and cervical ICA subocclusion were identified.

**Figure 2:**
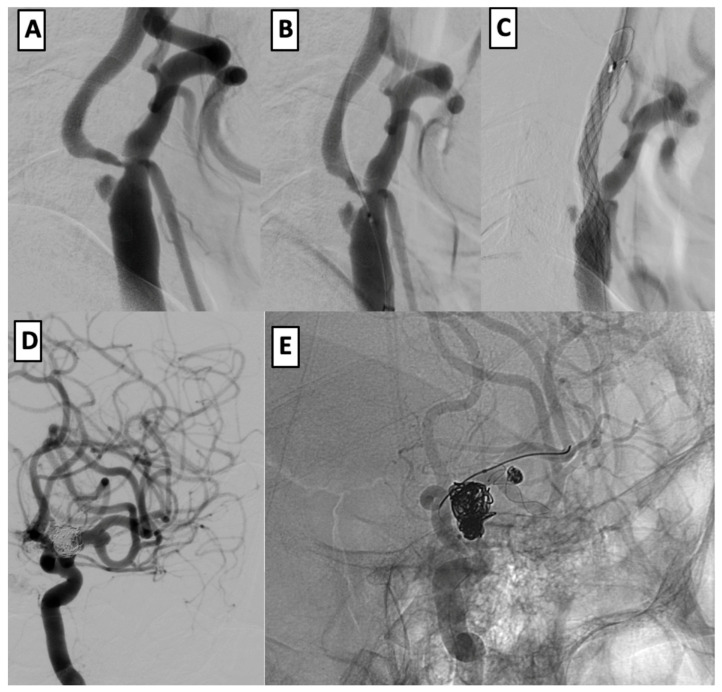
Endovascular management of dual intracranial aneurysms with cervical ICA stenosis. (**A**–**C**): angioplasty and CASPER stenting of the left cervical ICA after subocclusion and embolic protection. (**D**,**E**): coil embolization of left MCA and ICA aneurysms using half-T stenting technique with LEO stent (2.5 × 18 mm).

In a single-stage intervention, balloon angioplasty and stenting of the left cervical ICA were performed using a CASPER 9 × 20 × 143 mm stent, preceded by embolic protection and balloon pre-dilation. Subsequently, the MCA and ICA aneurysms were treated with stent-assisted coiling using a LEO stent (2.5 × 18 mm) and the half-T technique. The final angiography showed successful aneurysm exclusion and vessel patency. At the six-month follow-up, the patient had an mRS of 2 (Figure 2).

## 4. Discussion

This study adds to the growing body of the literature supporting the feasibility and safety of single-stage endovascular treatment for patients with coexisting intracranial aneurysms and extracranial arterial stenoses [2,4,7]. In the era of precision medicine, such combined interventions align with the shift toward individualized care that balances procedural efficiency with patient-specific anatomical and hemodynamic considerations [5,6].

All patients were followed for a uniform period of 6 months, capturing intermediate-term recovery and early complications such as in-stent thrombosis or aneurysm recurrence. While longer-term surveillance is needed to evaluate durability, this timeframe offers a reliable snapshot of initial outcomes.

In our cohort, smoking was the only factor significantly associated with the presence of multiple stenoses (*p* = 0.0191), reinforcing its role as a systemic atherosclerotic risk factor [8]. This finding aligns with the established literature on smoking’s adverse effects on endothelial integrity and vascular remodeling [9]. Other risk factors such as hypertension or ischemic heart disease were not independently predictive, likely reflecting sample size limitations.

**Figure 3:**
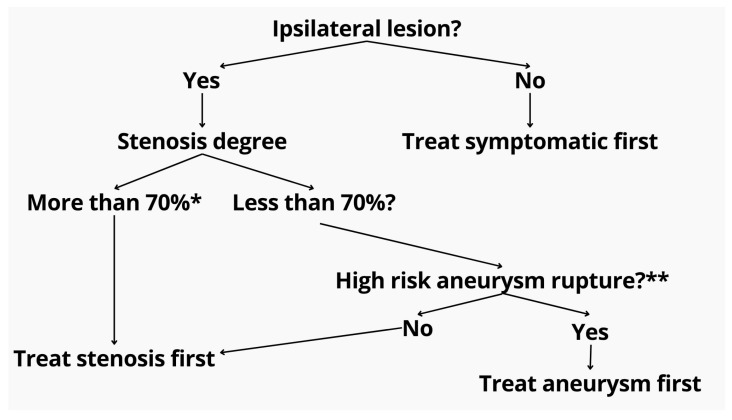
Proposed treatment algorithm for patients with coexisting extracranial stenosis and intracranial aneurysm. The initial step evaluates lesion ipsilaterality. If non-ipsilateral, the symptomatic pathology is prioritized. For ipsilateral lesions, the degree of stenosis is assessed. Stenoses exceeding 70% (as measured by NASCET criteria *) are treated first to ensure safe access and perfusion. If the stenosis is <70%, aneurysm rupture risk is evaluated using the PHASES score **. High-risk aneurysms are prioritized for embolization, while low-risk cases proceed with stenosis treatment. * North American Symptomatic Carotid Endarterectomy Trial (NASCET) criteria. ** Population, hypertension, age, size of aneurysm, earlier SAH, site of aneurysm (PHASES) score.

Based on our findings and the prior literature, we propose a decision-making algorithm for single-stage intervention, which prioritizes ipsilaterality, symptomatology, lesion size, and anatomical feasibility (Figure 3).

Our proposed algorithm (Figure 3) also incorporates real-world clinical reasoning suggested by experienced practitioners. Specifically, for symptomatic or >70% asymptomatic stenosis with an unruptured aneurysm—regardless of laterality—ICA stenting is prioritized before aneurysm embolization. The rationale includes the following: (1) reducing hypoperfusion risk during aneurysm navigation, (2) enabling stable catheter positioning distal to the stenosis, and (3) minimizing financial burden in low-resource settings by consolidating procedures and reducing repeated device use. Moreover, treating the aneurysm after stenting may mitigate the risk of pressure-related rupture, also known as normal perfusion breakthrough syndrome. In contrast, in cases of ruptured aneurysm with coexisting symptomatic or severe asymptomatic stenosis, the approach shifts toward initial angioplasty of the ICA to facilitate safe access, followed by aneurysm coiling. If a stent is required for the aneurysm, the ICA stenosis can be addressed concurrently. This sequence minimizes procedural delay in hemorrhagic cases while still preserving cerebral perfusion and catheter stability.

All patients were followed for a uniform period of six months, capturing intermediate-term recovery and procedural complications such as in-stent thrombosis or aneurysm recurrence. Although no patients in our cohort experienced these complications, we have contextualized this in the discussion by referencing the most commonly reported complications—groin hematoma, in-stent thrombosis, and distal embolization—and their incidence rates in the general population. The absence of these events in our group highlights the potential safety of the single-stage strategy, though the relatively small sample limits generalizability.

Balancing ischemic and hemorrhagic risk in these patients remains challenging. Historically, staged approaches like carotid endarterectomy followed by delayed aneurysm embolization were preferred [4,5,7], but they entail logistical and procedural drawbacks. Recent reports suggest that carefully selected patients may benefit from simultaneous interventions [2,8,9].

Case reports highlight the danger of treating carotid stenosis before securing aneurysms. Hartmann et al. [1] described a fatal post-stenting hemorrhage, while Pappada et al. [2] and Adams [5] emphasized the complexity of sequential decisions. Our study supports that, with structured planning, concurrent treatment is not only possible but safe.

Badruddin et al. [8] and Gallego Leon et al. [9] reported strong outcomes in similar cohorts. Our complication rate was low, and factors such as lesion laterality and stenosis length—rather than aneurysm size—were more closely associated with outcome.

We encountered two major complications: aneurysmal rebleeding post-stenting requiring decompressive craniectomy, and intraoperative rupture treated with nimodipine. These underscore the importance of risk stratification, especially in ruptured cases. Hartmann et al. [1] reinforced the theoretical risk of rebleed post-revascularization, validating strategies like coiling first or strict hemodynamic control.

The observed correlation between left-sided aneurysms and favorable outcome was unexpected. Given the complexity of left carotid access and symptomatic burden of left hemispheric strokes [10], this may reflect anatomical variance in our cohort.

These findings suggest that individualized anatomy-driven strategies—potentially aided by AI-based imaging—can enhance procedural outcomes. The lack of correlation between mRS and aneurysm size or stenosis degree suggests that vascular morphology may carry more prognostic value than previously assumed.

Procedurally, our series included patients with ipsilateral, contralateral, symptomatic, and ruptured lesions—all treated in one session. This broad applicability supports single-stage endovascular intervention as a viable alternative to staged approaches in experienced centers [2,4,7,11].

In most cases, CAS was performed first to secure proximal access and reduce embolic load during aneurysm navigation [2,5,12]. When feasible, aneurysm coiling preceded stenting in morphologically unstable lesions. Such flexibility, guided by real-time angiography and preoperative imaging, reflects evolving neurointerventional strategy [6,13].

One high-risk case involved an SAH patient with critical ICA stenosis and impaired perfusion, treated emergently with stenting before embolization. Despite concerns over dual antiplatelet therapy (DAPT) in hemorrhagic states, this decision prioritized cerebral perfusion. Emerging data support cautious DAPT use in such settings [14,15], though we remain conservative and defer stenting when possible in acute SAH.

### Limitations

This study has several limitations that must be acknowledged. First, the retrospective and single-center design introduces inherent risks of selection bias and limits the generalizability of the findings. Although our patient population reflects real-world clinical complexity, the absence of randomization restricts the ability to draw causal inferences regarding the superiority of single-stage intervention over staged or conservative approaches.

Second, the relatively small sample size (n = 47) constrains the statistical power, particularly in subgroup analyses such as aneurysm location or lesion-specific characteristics. Larger multicenter cohorts would provide more robust evidence to validate the identified associations—such as the impact of stenosis length and aneurysm laterality on functional outcome.

Third, the follow-up duration, with a minimum of three months, may not be sufficient to capture late complications such as in-stent restenosis, delayed aneurysm recurrence, or long-term neurocognitive deficits. A longer follow-up period is essential for assessing the durability and neurological sequelae of the treatment.

Fourth, while the modified Rankin Scale (mRS) is a widely accepted outcome measure, it may not adequately reflect subtle cognitive or functional impairments, particularly in patients with anterior circulation lesions. Future research should incorporate more detailed neuropsychological and quality-of-life assessments.

Finally, the treatment of multiple aneurysms in a single session alongside extracranial stenosis represents a complex and underexplored subgroup. We plan to collect more cases and conduct focused analysis to better understand outcomes in this patient population.

Despite these limitations, our study offers valuable insights into the evolving paradigm of precision-guided single-session endovascular therapy in complex cerebrovascular disease.

## 5. Conclusions

This study supports the feasibility and safety of single-stage endovascular treatment in patients with concurrent intracranial aneurysms and extracranial arterial stenoses. Our findings suggest that lesion-specific factors—particularly aneurysm laterality and stenosis length—may influence functional outcomes more significantly than aneurysm size or stenosis degree. Smoking emerged as a strong independent predictor of multiple stenoses, underscoring the importance of modifiable risk factor management in cerebrovascular patients.

Future prospective multicenter studies with longer follow-up and expanded functional outcome measures are warranted to refine treatment algorithms and establish evidence-based protocols for this complex patient population.

## Figures and Tables

**Table 1:** Patient Characteristics and Treatment Details for Concurrent Intracranial Aneurysms and Arterial Stenoses.

Patient #	Age	Gender	Presenting Symptoms	Aneurysm (#) and Localization	Stenosis Localization	Ipsilateral Lesion?	Aneurysm Size	Stenosis Length	Stenosis Degree	Stents Used	mRS Before	mRS FU
1	67	m	Persistent headache	(2) A1 left, right MCA bif	M2 left	Yes	Small	20 mm	70%	Ultimaster 3.5× 9;	2	2
2	70	m	Left cerebellar stroke, persistent headache, sensorineural hearing loss	(1) A1 left	V1 left, Left ICA supraclinoid	Yes	Small	15 mm	80%	Destiny 3.0 × 15	1	1
3	75	m	Persistent headache	(1) Right ICA, supraclinoid	V1 right	Yes	Small	15 mm	70%	Ultimaster 3.5 × 9;	2	2
4	72	m	Persistent headache	(1) Right MCA bif	V1 left, V1 right	Yes	Small	30 mm	70%	Ultimaster Nagomi 2.5 × 12; Orsiro 3.5 × 26	1	1
5	65	f	Right frontal and parietal strokes with left-sided hemiparesis	(1) Right MCA bif	Left MCA bif	No	Small	15 mm	70%	LVIS EVO 3.0 × 18	3	3
6	65	m	Left occipital stroke	(1) Right ICA, ophthalmic	V1 left	No	Small	10 mm	70%	Ultimaster 4.0 × 9;	1	1
7	45	f	Persistent headache	(1) Right ICA, supraclinoid	Right ICA, cervical	Yes	Medium	35 mm	80%	X.ACT 8-6 × 30; Carotid WALLSTENT 7–30 × 135	1	1
8	40	f	Persistent headache	(2) Right ICA, supraclinoid, Left ICA ophthalmic	Left ICA supraclinoid	Yes	Small	20 mm	70%	pEGASUS 4.5 × 20	1	1
9	72	f	Persistent headache	(1) Left ICA, communicant	BA	N/A	Small	20 mm	80%	CREDO 5.0 × 20	1	1
10	60	m	Persistent headache	(2) Left M2, left ICA supraclinoid	Right ICA, supraclinoid, cavernous	No	Small	30 mm	80%	Credo 5.0 × 25; Ultimaster 4.0 × 12	2	2
11	69	m	Right frontal and parietal strokes with left-sided hemiparesis	(1) Right ICA, cavernous	Right ICA, supraclinoid	Yes	Small	20 mm	70%	CREDO 4.0 × 20	2	2
12	57	m	Persistent headache	(2) Left ICA, communicant, Left MCA bif	Left ICA, cervical	Yes	Medium	40 mm	99%	X.ACT 10-10 × 30 CASPER 9 × 20 × 143	2	2
13	38	m	Right cerebellar stroke	(1) Left MCA bif	V1 right	No	Small	20 mm	80%	Ultimaster 3.5 × 12	2	1
14	64	f	Left frontal stroke with right-sided hemiparesis	(1) Left MCA bif	Left М2	Yes	Small	20 mm	99%	CREDO 4.0 × 20	2	2
15	60	f	Persistent headache	(1) Right ICA, communicant	Right ICA supraclinoid	Yes	Small	25 mm	75%	CASPER 7 × 30 × 143	1	1
16	43	m	Ruptured aneurysm, left-sided hemiparesis	(3) Right MCA bif, left MCA bif, left ICA choroidal	V1 left, left ICA cervical, right ICA, cervical	Yes	Medium	50 mm	99%	Ultimaster 2.75–9; BioMatrix 3.5 × 9; Wallaby 3.0 × 40; Wallaby 2.5 × 40	3	3
17	56	f	Persistent headache, sensorineural hearing loss	(1) Left MCA bif	Left ICA cervical, right ICA, cervical	Yes	Medium	40 mm	75%	CGuard 10 × 40; CGuard 9 × 30; LEO 2.5 × 25	1	1
18	59	m	Ruptured aneurysm, right parietal strokes, left-sided hemiparesis	(1) BA fusiform	Right ICA, cervical	N/A	Fusiform	25 mm	80%	Mozec 4.5 × 23; СGuard 9 × 40; LEO + 4.5 × 75	3	3
19	68	f	Persistent headache	(1) Right ICA, supraclinoid	V1 left	No	Small	10 mm	90%	Ultimaster Terumo 4.0 × 9.0	1	1
20	57	f	Left temporal stroke	(1) Left MCA bif	Left M1-M2	Yes	Medium	20 mm	70%	Acclino flex 5.0 × 20	2	2
21	66	m	Left frontal stroke	(1) Right MCA bif	Left ICA, cervical	No	Medium	30 mm	70%	Protege 8 × 6–30	1	1
22	64	f	Persistent headache	(1) Right M1	Right ICA, cervical	Yes	Small	30 mm	70%	Protege 8 × 6–30	1	1
23	69	m	Left frontal stroke with right-sided hemiparesis	(1) BA bif	Left ICA, cervical	N/A	Small	30 mm	85%	Protege 8 × 6–30	3	3
24	59	f	Right occipital, cerebellar, and basal ganglia strokes	(1) Acom	V1 Right	N/A	Small	30 mm	95%	AclinoFlex 3.0 × 15	4	4
25	69	m	Right frontal stroke	(1) Left MCA bif	Right ICA cervical	No	Small	40 mm	80%	Protege 8 × 6–40	2	2
26	66	m	Ruptured aneurysm	(1) Left MCA bif	Right ICA supraclinoid	No	Small	30 mm	85%	Protege 8 × 6–30	2	2
27	58	f	Persistent headache	(1) Right ICA supraclinoid	Left ICA, cervical	No	Small	30 mm	80%	Protege 7 × 10–30	2	2
28	61	m	Left occipital and cerebellar strokes	(1) Right M2	V1 Left	No	Small	30 mm	80%	Ultimaster 4.0 × 9; Ultimaster 2.5 × 9; Ultimaster 3.0 × 9	2	2
29	70	f	Right frontal and parietal strokes	(1) Right ICA supraclinoid	Right ICA, cervical	Yes	Small	40 mm	70%	Protege 8-6 × 30	2	2
30	77	f	Right frontal and parietal strokes	(1) Right MCA bif	Right ICA, cervical	Yes	Small	40 mm	85%	Protege 8-6 × 40	2	2
31	65	m	Ruptured aneurysm, left frontal and parietal strokes	(2) Left ICA, ophtalmic, terminus	Left ICA, cervical	Yes	Small	40 mm	80%	Protege 8-6 × 40; LEO 2.0 × 18	2	2
32	75	f	Ruptured aneurysm, right frontal and parietal strokes	(2) Right ICA, communicant, right MCA bif	Right ICA, cervical, left ICA ophtalmic	Yes	Medium	25 mm	70%	Protege 6 × 8–30	3	3
33	56	f	Left temporal stroke	(1) Acom	Left ICA, cervical	N/A	Medium	30 mm	70%	Protege 8-6 × 30	2	2
34	60	f	Ruptured aneurysm, right frontal and parietal strokes	(1) Right MCA bif	Right ICA, cervical	Yes	Small	15 mm	85%	AclinoFlex 3.0 × 15	3	5
35	59	f	Ruptured aneurysm, multiple strokes	(6) Acom, right MCA bif, right ICA ophtalmic, communicant, supraclinoid, left ICA ophtalmic	Right ICA, cervical	Yes	Small	30 mm	80%	Protege 8 × 6–30	3	3
36	55	m	Ruptured aneurysm	(1) Right ICA, communicant	Right ICA cervical	Yes	Medium	30 mm	70%	Protege 8 × 6–30	2	2
37	56	f	Ruptured aneurysm	(1) BA bif	Left ICA cavernous	N/A	Small	30 mm	75%	Orsiro 3.50 × 1; Solitaire AB 6 × 30	2	2
38	63	m	Left frontal stroke	(1) Left MCA bif	Left ICA, cervical	Yes	Small	40 mm	80%	Protege 6 × 8–40 (2)	2	2
39	40	f	Persistent headache	(1) Left ICA, ophtalmic	Left ICA, cervical	Yes	Medium	40 mm	70%	Protege 8 × 6–40; Protege 8 × 6–30	2	2
40	59	f	Right occipital and cerebellar strokes	(1) Right ICA, communicant	V1 right	No	Medium	40 mm	80%	Protege 6 × 8–40	3	4
41	64	f	Left cerebellar stroke	(1) V1 left	V1 left	Yes	Small	10 mm	70%	Resolute ONYX 2.0 × 8	2	2
42	53	m	Right frontal stroke	(2) Acom, left ICA, cavernous	Left ICA, cervical, V1 right, V1 left	Yes	Medium	30 mm	70%	Protege 6 × 8–40	2	2
43	82	f	Persistent headache	(1) Right ICA, cavernous	Right ICA, cervical	Yes	Fusiform	30 mm	70%	Protege 8 × 6–30, Pipeline with Shield 4.5 × 25	2	2
44	63	f	Repeated pontine ischemia, Millard–Gubler syndrome	(1) BA bif	V1 left	N/A	Medium	15 mm	80%	Ultimaster 3.5 × 15	2	2
45	66	m	Right frontal and parietal strokes	(1) Right MCA bif	Right ICA, cervical	Yes	Small	30 mm	85%	CASPER 7-30 × 143	2	2
46	67	f	Persistent headache	(1) Right ICA, ophthalmic	Right ICA, cervical	Yes	Small	40 mm	90%	Protege 6 × 8–40	1	1
47	30	f	Left occipital and cerebellar strokes	(1) V2 right	V3-V4 left	Yes	Small	15 mm	80%	XIENCE Xpedition 4 × 12	3	3

# stands for a number

**Table 2:** Association between clinical factors and occurrence of multiple stenoses and multiple aneurysms.

	One Stenosis Localization	Multiple Stenosis Localization	Total	*p*-Value Fisher’s
Smoking				
No	28 (71.79%)	2 (25.00%)	30	0.0191
Yes	11 (28.21%)	6 (75.00%)	17	
AH				
AH1	3 (7.69%)	0 (0.00%)	3	0.1217
AH2	3 (7.69%)	3 (37.50%)	6	
AH3	33 (84.62%)	5 (62.50%)	38	
Diabetes				
No	31 (79.49%)	5 (62.50%)	36	0.3673
Yes	8 (20.51%)	3 (37.50%)	11	
IHD				
No	13 (33.33%)	0 (0.00%)	13	0.0855
Yes	26 (66.67%)	8 (100.00%)	34	
Obesity				
Non-obese	29 (74.36%)	7 (87.50%)	36	0.6593
Obese	10 (25.64%)	1 (12.50%)	11	
	**One Aneurysm**	**Multiple Aneurysms**	**Total**	***p*** ** -V alue Fisher’s**
Smoking				
No	26 (68.42%)	4 (44.44%)	30	0.2516
Yes	12 (31.58%)	5 (55.56%)	17	
AH				
AH1	1 (2.63%)	2 (22.22%)	3	0.1201
AH2	5 (13.16%)	1 (11.11%)	6	
AH3	32 (84.21%)	6 (66.67%)	38	
Diabetes				
No	28 (73.68%)	8 (88.89%)	36	0.6631
Yes	10 (26.32%)	1 (11.11%)	11	
IHD				
No	11 (28.95%)	2 (22.22%)	13	1
Yes	27 (71.05%)	7 (77.78%)	34	
Obesity				
Non-obese	28 (73.68%)	8 (88.89%)	36	0.6631
Obese	10 (26.32%)	1 (11.11%)	11	

**Table 3:** Relationship between aneurysm/stenosis localization and mRS outcome.

	mRS 1–2	mRS 3–6	Total	*p*-Value Fisher’s
Aneurysm side				
Left	14 (37.84%)	0 (0.00%)	14	0.019
Bilateral	6 (16.22%)	5 (50.00%)	11	
Right	17 (45.95%)	5 (50.00%)	22	
Aneurysm arteries				
ACA	1 (2.70%)	0 (0.00%)	1	0.0761
Acom	1 (2.70%)	1 (10.00%)	2	
BA	2 (5.41%)	2 (20.00%)	4	
ICA	15 (40.54%)	1 (10.00%)	16	
MCA	13 (35.14%)	2 (20.00%)	15	
Bilateral	4 (10.81%)	3 (30.00%)	7	
VA	1 (2.70%)	1 (10.00%)	2	
Stenosis side				
Left	17 (45.95%)	3 (30.00%)	20	0.7017
Bilateral	5 (13.51%)	2 (20.00%)	7	
Right	15 (40.54%)	5 (50.00%)	20	
Stenosis arteries				
BA	1 (2.70%)	0 (0.00%)	1	0.9497
ICA	22 (59.46%)	5 (50.00%)	27	
MCA	3 (8.11%)	1 (10.00%)	4	
Multiple	3 (8.11%)	1 (10.00%)	4	
VA	8 (21.62%)	3 (30.00%)	11	
The stenosis side and aneurysm side coincide				
N/A	4 (10.81%)	3 (30.00%)	7	0.3108
No	9 (24.32%)	2 (20.00%)	11	
Yes	24 (64.86%)	5 (50.00%)	29	

**Table 4:** Impact of aneurysm size, stenosis length, and degree on mRS outcome.

	mRS 1–2	mRS 3–6	Total	*p*-Value Fisher’s
Aneurysm Size				
Fusiform	1 (2.70%)	1 (10.00%)	2	0.4291
Medium	10 (27.03%)	3 (30.00%)	13	
Small	26 (70.27%)	6 (60.00%)	32	
Stenosis length				
10 mm	3 (8.11%)	0 (0.00%)	3	0.0469
15 mm	3 (8.11%)	3 (30.00%)	6	
20 mm	7 (18.92%)	0 (0.00%)	7	
25 mm	1 (2.70%)	2 (20.00%)	3	
30 mm	13 (35.14%)	3 (30.00%)	16	
35 mm	1 (2.70%)	0 (0.00%)	1	
40 mm	9 (24.32%)	1 (10.00%)	10	
50 mm	0 (0.00%)	1 (10.00%)	1	
Stenosis degree				
70	16 (43.24%)	2 (20.00%)	18	0.2588
75	3 (8.11%)	0 (0.00%)	3	
80	11 (29.73%)	4 (40.00%)	15	
85	3 (8.11%)	2 (20.00%)	5	
90	2 (5.41%)	0 (0.00%)	2	
95	0 (0.00%)	1 (10.00%)	1	
99	2 (5.41%)	1 (10.00%)	3	

## Data Availability

The data supporting the findings of this study are available from the corresponding author upon reasonable request. Due to patient privacy regulations and institutional policy, individual-level data cannot be publicly shared but may be provided in anonymized form for academic and research purposes.

## Abbreviations

The following abbreviations are used in this manuscript:

ACA	Anterior Cerebral Artery
Acom	Anterior Communicating Artery
BA	Basilar Artery
CAS	Carotid Artery Stenting
CTA	Computed Tomography Angiography
DAPT	Dual Antiplatelet Therapy
DSA	Digital Subtraction Angiography
DWI	Diffusion-Weighted Imaging
ICA	Internal Carotid Artery
IDH	Isocitrate Dehydrogenase
IHD	Ischemic Heart Disease
MCA	Middle Cerebral Artery
mRS	Modified Rankin Scale
MRI	Magnetic Resonance Imaging
SAH	Subarachnoid Hemorrhage
TIA	Transient Ischemic Attack
VA	Vertebral Artery
